# Applicability of ultrasonography for detection of marginal sinus placenta previa

**DOI:** 10.1097/MD.0000000000024253

**Published:** 2021-01-08

**Authors:** Hiroki Ishibashi, Morikazu Miyamoto, Hiroshi Shinmoto, Shigeyoshi Soga, Hideki Iwahashi, Soichiro Kakimoto, Hiroko Matsuura, Takahiro Sakamoto, Taira Hada, Rie Suzuki, Masashi Takano

**Affiliations:** a Department of Obstetrics and Gynecology; b Department of Radiology, National Defense Medical College Hospital, Tokorozawa, Saitama, Japan.

**Keywords:** magnetic resonance imaging, marginal sinus placenta previa, placenta previa, ultrasonography

## Abstract

This study aimed to examine whether marginal sinus placenta previa, defined as when the marginal sinus just reaches the internal cervical os and placental parenchyma might be >2 cm from the internal cervical os, can be diagnosed using ultrasonography (US). We identified the placenta previa cases that underwent both US and magnetic resonance imaging (MRI) between April 2010 and December 2018 at our institution. The diagnostic discrepancies for marginal sinus placenta previa between US and MRI were examined retrospectively. Of the 183 cases of placenta previa, 28 (15.3%) cases were diagnosed as marginal sinus placenta previa using MRI. Among them, 18 cases (64.3%) could also be diagnosed using US. The sensitivity and specificity of the diagnosis of marginal sinus placenta previa using US were 64.3% and 92.9%, respectively. A change in US diagnosis occurred in 10 (35.7%) cases, all of which were diagnosed with low-lying placenta previa or marginal placenta previa and did not develop any serious miserable obstetrical outcomes. In conclusion, the diagnostic accuracy of US for detecting marginal sinus placenta previa was not significant. MRI examination may be required to accurately categorize the types of placenta previa.

## 1 Introduction

Placenta previa occurs in approximately 0.4% of all live births and is a major cause of maternal hemorrhage and morbidity.^[1,2]^ The prevalent diagnostic method used for placenta previa is transvaginal ultrasonography (US) in the third trimester up to 32 gestational weeks.^[3]^ In addition, magnetic resonance imaging (MRI) is an effective alternative method.^[4]^

Previously, we reported the clinical significance of MRI-diagnosed marginal sinus placenta previa**—**defined as placenta previa whose marginal sinus just reaches the internal cervical os and when the placental parenchyma may be >2 cm from the internal cervical os.^[5,6]^ Marginal sinus placenta previa is a mild type of placenta previa in the clinical setting because the intra- and post-operative hemorrhage is not statistically different from those patients with minor placenta previa, including low-lying placenta previa and marginal placenta previa. However, it is unclear whether marginal sinus placenta previa can be diagnosed using US.

Therefore, this study aimed to investigate whether US can diagnose marginal sinus placenta previa.

## 2 Methods

Singleton pregnancy cases diagnosed with placenta previa using both US and MRI at our institution between April 2010 and December 2018 were identified. Cases without medical records were excluded.

Maternal histories and intraoperative information were obtained from the medical charts and operative records. In all cases, the diagnosis of placenta previa was made by experienced obstetricians and radiologists based on US and MRI examinations at 28 to 34 gestational weeks. The diagnosis of placenta previa by both US and MRI was performed at the same time.

**Figure 1:**
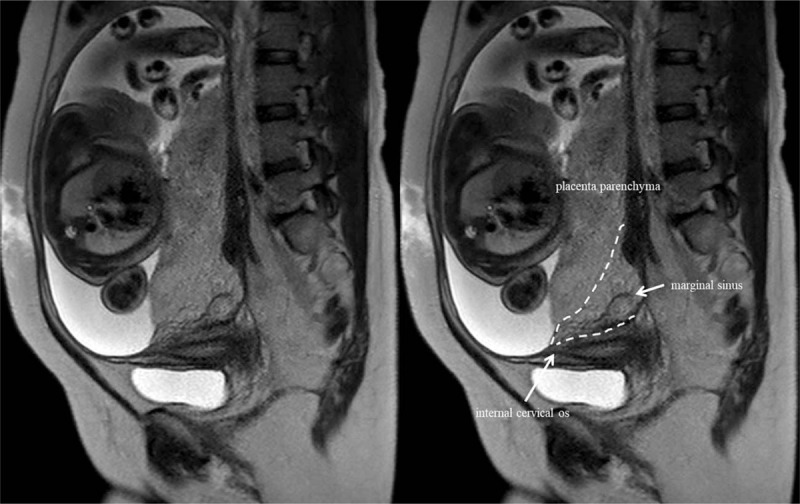
Representative magnetic resonance imaging of marginal sinus placenta previa (sagittal T2-weighted imaging).

Pelvic MRI examination was performed using a 1.5 tesla scanner (Ingenia, Philips Healthcare, Eindhoven, Netherlands). They were imaged in the supine position using a 32-channel phased-array coil. MRI evaluation of the placenta without the use of gadolinium was performed in all cases to determine the accurate placental location, type of previa, and placental adhesion. The maternal pelvis was scanned in axial, sagittal, and coronal respiratory-triggered single-shot fast spin echo sequence (repetition time/echo time = 1500/100 ms, 6 mm slice thickness with 1 mm gap, 304 × 276 (zero-filled interpolation 512) matrices) and sagittal T1-weighted fast-spin echo sequence (repetition time/echo time = 253/4.6 ms, 6 mm slice thickness with 1 mm gap, 240 × 214 (zero-filled interpolation 352) matrices). A representative magnetic resonance image of marginal sinus placenta previa is shown in Figure 1.

**Figure 2:**
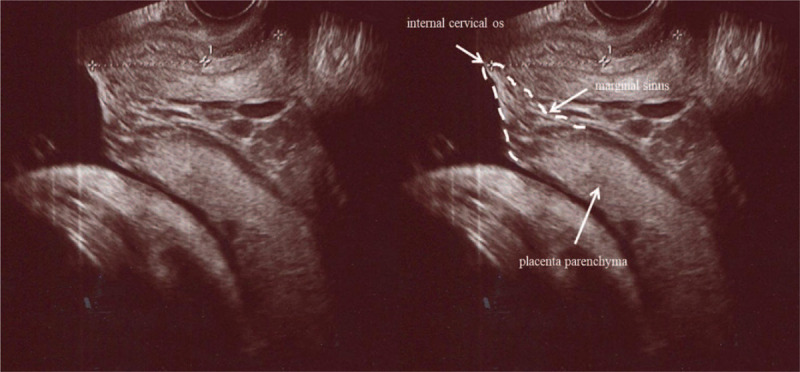
Representative transvaginal ultrasonography image of marginal sinus placenta previa (sagittal grayscale imaging).

During the 2D transvaginal US examination, observation of the uterine cervix, uterine lower segment, and placenta were performed using US equipment (Voluson E-6 or E-10; GE Medical Systems Kretztechnik, Zipf, Austria; mechanical transvaginal transducer, 7.5 MHz). The US analysis was performed with the patient in the lithotomy position with an empty bladder. The cervical canal from the external to the internal cervical os and placenta was clearly visualized. The placental edge near the internal cervical os was determined as the outer edge of the placenta parenchyma or marginal sinus with color Doppler. A representative US image of a marginal sinus placenta previa is shown in Figure 2.

In our study, placenta previa has been classified into 3 groups: marginal sinus placenta previa, minor placenta previa, and major placenta previa. The definition of marginal sinus placenta previa was when the placental marginal sinus of placenta previa just reaches the internal cervical os and when the placental parenchyma is >2 cm from the internal cervical os.^[6]^ If the edge of the placenta covered the internal cervical os, it was diagnosed as a major type, which includes partial and total placenta previa. If the edge of the placenta did not cover the internal cervical os, and was located in the lower uterine segment, it was diagnosed as a minor type, which includes low-lying and marginal placenta previa.^[7]^ The placental location was determined by whether over 50% of the placenta was located on the anterior or posterior wall using US and MRI.

Statistical analysis was performed using JMP 14.0 software (SAS Institute Inc., Cary, NS, USA). The sensitivity, specificity, positive predictive value, and negative predictive value of the diagnosis of marginal sinus placenta previa using US were calculated. The 95% confidence interval for the estimated parameter was calculated based on a constant chi-square confidence interval.

This study was approved by the ethical committee of our hospital at the National Defense Medical College, Tokorozawa, Japan (approval number: 3097). Because our study was retrospective analysis, informed consent did not be obtained. However, the chance of refusal to participate our study was put on the website of our hospital and we ensure its chance.

## 3 Results

During the study period, 183 cases were included in this study. The mean gestational age at diagnosis using US and MRI was 33 gestational weeks. There were 28 of 183 (15.3%) cases with marginal sinus placenta previa diagnosed using MRI. Among them, there were 18 cases (64.3%) diagnosed with marginal sinus placenta previa using US. The sensitivity, specificity, positive predictive value, and negative predictive value of US examination were 64.3%, 92.9%, 62.1%, and 93.5%, respectively (Table 1).

**Table 1:** Sensitivity, specificity, positive predictive value, negative predictive value, and accuracy of the prediction of marginal sinus placenta previa using ultrasonography.

	Sensitivity (95% CI)	Specificity (95% CI)	Positive predictive value (95% CI)	Negative predictive value (95% CI)
Prediction of marginal sinus placenta previa using US	64.3% (49.2–76.4)	92.9% (90.2–95.1)	62.1% (47.5–73.7)	93.5% (90.8–95.7)

Ten (35.7%) cases that were not diagnosed with marginal placenta previa using US are summarized in Table 2. All these cases had posterior placenta. No patient received additional hemostatic procedures, including allogeneic blood transfusion, and none of these patients developed any miserable obstetrical outcomes.

**Table 2:** Clinical characteristics of 10 cases with false positive marginal sinus placenta previa diagnosis using ultrasonography.

Age	MRI findings	Placental location	Antenatal bleeding	GA at delivery (week)	Emergency CS	Intraoperative hemorrhage (ml)	Postoperative hemorrhage (ml)	PAS	Additional hemostatic procedures	Allogenic blood transfusion
35	Minor placenta previa	Posterior	Negative	37+1	Negative	1044	40	Negative	Negative	Negative
41	Minor placenta previa	Posterior	Negative	36+6	Negative	1856	110	Negative	Negative	Negative
32	Minor placenta previa	Posterior	Negative	36+6	Negative	410	45	Negative	Negative	Negative
31	Minor placenta previa	Posterior	Negative	37+2	Negative	520	25	Negative	Negative	Negative
36	Minor placenta previa	Posterior	Negative	37+3	Negative	598	22	Negative	Negative	Negative
36	Minor placenta previa	Posterior	Negative	37+5	Negative	380	83	Negative	Negative	Negative
39	Minor placenta previa	Posterior	Negative	37+2	Negative	698	126	Negative	Negative	Negative
31	Minor placenta previa	Posterior	Negative	37+6	Negative	558	96	Negative	Negative	Negative
33	Minor placenta previa	Posterior	Negative	37+1	Negative	847	30	Negative	Negative	Negative
40	Minor placenta previa	Posterior	Negative	37+2	Negative	880	50	Negative	Negative	Negative

In 28 cases diagnosed as marginal sinus placenta previa using MRI, 1 patient (3.6%) had anterior placenta, and 27 cases (96.4%) had posterior placenta. One case with anterior placenta was also diagnosed as marginal sinus placenta previa using US, while 17/27 cases (63.0%) were diagnosed using US in the cases with posterior placenta.

## 4 Discussion

The pivotal management for placenta previa includes the preparation of massive hemorrhage such as hemostatic procedures (e.g., balloon tamponade, uterine artery embolization, hysterectomy) and allogenic blood transfusion.^[8,9]^ Our previous study using MRI revealed there were no patients in marginal sinus placenta previa who received allogenic blood transfusion and uterine artery embolization for massive hemorrhage compared with those in major placenta previa.^[5]^ Thus, the clinical significance of marginal sinus placenta previa was consistent with that of minor placenta previa and might be a milder type than major placenta previa.^[5]^ In addition, there were 4/27 (14.8%) cases with marginal sinus placenta previa diagnosed among those with partial placenta previa before re-evaluation.^[5]^ Therefore, the classification including marginal sinus placenta previa might identify cases with low risk of massive hemorrhage among cases with major placenta previa.

The prevalent diagnostic method of placenta previa is transvaginal US in the second or third trimester.^[3]^ Although MRI examination was used as an auxiliary modality, its cost is higher than that of US.^[10]^ Therefore, if the placenta previa could be diagnosed accurately by US, it might be the preferred diagnostic method.

Surprisingly, the current study shows that the sensitivity of US for the diagnosis of marginal sinus placenta previa was not so high. One reason for this low sensitivity might be the placental location. It was more difficult to visualize posterior placenta previa than anterior placenta previa by transvaginal US.^[11]^ In the current study, all cases exhibiting a diagnostic discrepancy between US and MRI had posterior placenta. Hence, MRI examination can visualize both anterior and posterior placenta previa more clearly than US.^[12]^ Therefore, diagnosis by US examination to categorize the types of posterior placenta previa has limitations, and an MRI examination might be necessary. Another reason might be the fetal head pressure on the lower uterine segment. US assessment of the lower uterine segment has been reported to be affected by fetal head compression.^[13]^ Furthermore, since the median timing of diagnosis was 32 gestational weeks in our study, the fetus sufficiently grew to compress the marginal sinus, thus decreasing the marginal sinus in US images. Therefore, it was difficult to diagnose marginal sinus placenta previa using US.

Our study showed that all cases that were not diagnosed with marginal sinus placenta previa by US had minor placenta previa, and they did not develop any adverse clinical outcomes. Generally, major placenta previa is associated with higher morbidity than minor placenta previa.^[14]^ Therefore, misdiagnosis did not induce major clinical problems, such as massive hemorrhage. However, MRI examination might be required to enhance the diagnostic accuracy for the types of placenta previa.

There were several limitations to this study. This study was a retrospective and small case study. US could not accurately diagnose marginal sinus placenta previa in this study. Hence, further studies are needed to examine this problem.

In conclusion, the diagnostic accuracy for marginal sinus placenta previa with US was not so high. MRI examination might be required to accurately diagnose and classify the types of placenta previa.

## Acknowledgments

We would like to thank Editage for help with language editing of the manuscript.

## Author contributions

**Conceptualization:** Hiroki Ishibashi, Morikazu Miyamoto, Masashi Takano.

**Data curation:** Hiroki Ishibashi, Morikazu Miyamoto, Masashi Takano.

**Formal analysis:** Hiroki Ishibashi, Hiroshi Shinmoto, Shigeyoshi Soga.

**Funding acquisition:** Hiroki Ishibashi.

**Investigation:** Hiroki Ishibashi, Hideki Iwahashi, Soichiro Kakimoto.

**Methodology:** Hiroki Ishibashi, Hiroko Matsuura, Takahiro Sakamoto.

**Project administration:** Hiroki Ishibashi.

**Resources:** Taira Hada.

**Supervision:** Rie Suzuki.

**Validation:** Morikazu Miyamoto.

**Visualization:** Morikazu Miyamoto.

**Writing – original draft:** Hiroki Ishibashi

**Writing – review & editing:** Morikazu Miyamoto, Masashi Takano
